# Differential Efficacy of Neurofeedback in Children with ADHD Presentations

**DOI:** 10.3390/jcm8020204

**Published:** 2019-02-07

**Authors:** Marisol Cueli, Celestino Rodríguez, Paloma Cabaleiro, Trinidad García, Paloma González-Castro

**Affiliations:** Department of Psychology, University of Oviedo, Plaza Feijoo s/n, 33003 Oviedo (Asturias), Spain; cuelimarisol@uniovi.es (M.C.); cabaleiro@uniovi.es (P.C.); garciatrinidad@uniovi.es (T.G.); mgcastro@uniovi.es (P.G.-C.)

**Keywords:** ADHD, presentations, neurofeedback, cortical activation, intervention

## Abstract

Training in neurofeedback (NF) reduces the symptomatology associated with attention deficit with hyperactivity disorder (ADHD). However, ADHD differs in terms of the type of presentation, i.e., inattentive (ADHD-I), impulsive/hyperactive (ADHD-HI), or combined (ADHD-C). This study examines the efficacy of NF in ADHD presentations. Participants were 64 students (8–12 years old). Cortical activation, executive control, and observed symptomatology by parents were assessed. Results indicated that ADHD-C and ADHD-HI demonstrated greater improvements than ADHD-I. It was concluded that this kind of training produces an improvement and that it is necessary to explore it further in terms of the protocol used.

## 1. Introduction

Attention deficit with hyperactivity disorder (ADHD) is understood as a persistent pattern of inattentive, restless, and impulsive behavior which is more frequent and severe than that typically observed in subjects at a similar stage of development [1]. Its prevalence is estimated to be 5.9%–7.1% in childhood and adolescence, and 5% in adults [2]. The new classification of the Diagnostic and Statistical Manual of Mental Disorders (DSM), 5th edition (DSM-5), [1] included ADHD as a neurodevelopmental disorder and has replaced the differentiation between subtypes by types of presentation (predominantly hyperactive/impulsive, predominantly inattentive, and combined presentation) [3].

Given the symptomatology of ADHD and its high prevalence rates [4,5], it is important for researchers to analyze the efficacy of the different treatments and interventions aimed at inattention, hyperactivity, and impulsivity, such as medication, neurofeedback, and behavioral treatments. This study focuses specifically on the efficacy of neurofeedback in children with a diagnosis of hyperactive/impulsive, inattentive, and combined ADHD. Currently, stimulant medication and behavioral therapy are the most commonly applied treatments for ADHD [6], although recent large-scale studies and meta-analyses have demonstrated their limitations [6,7,8]. 

Some authors have noted that stimulant medication is effective in reducing ADHD symptoms in 70%–80% of children suffering from ADHD [9,10]. Thus, about a quarter of adolescents with ADHD do not sufficiently benefit from standard treatment with stimulant medication [9]. Hodgson, Hutchinson, and Denson [11] carried out a meta-analysis of behavioral treatments for ADHD, comparing the efficacy of seven non-pharmacological interventions (behavior modification, neurofeedback therapy, multimodal psychosocial treatment, school-based programs, working memory training, parental training, and self-monitoring). Their results showed that behavior modification and neurofeedback treatments were most supported by the evidence. In this sense, in the past few years, some studies have stated the effectiveness of neurofeedback training with the objective of increasing levels of cortical activation [12,13,14]. Neurofeedback (NF) is aimed at teaching or improving self-regulation [6,15], and it is based on classical conditioning principles (learning new behaviors through the process of association) applied to the electroencephalogram (EEG) [6]. Training in NF emerged as an intervention for stimulating cortical activation [16,17], particularly in disorders associated with inattention, and deficits in self-regulation and control skills such as ADHD [18]. Previous studies have demonstrated an increase in activation by NF training, not only due to immediate feedback (visual and auditory) provided by the instrument, but also due to the establishment of new neural pathways and connections [13,19]. For example, González-Castro, Cueli, Rodríguez, García, and Álvarez [20] compared the efficacy of NF and pharmacological support in 131 students. Their results indicated that the combined group (NF and pharmacological support) benefited more and that the NF group improved to a greater extent in executive control (measured by a continuous performance task) than the pharmacological support group.

Clinicians frequently use three types of protocols for NF training in ADHD [12]: (1) a conventional protocol to reduce inattention and impulsivity, which consists of operant suppression of theta activity and enhancement of beta activity [21,22]; (2) a protocol to reduce hypermotoric symptoms and enhance sensorimotor rhythm (SMR) [18,23]; and (3) a protocol referred to electrophysiological evidence of altered slow cortical potentials (SCPs) in ADHD. This third protocol is aimed at modifying SCPs in order to regulate cortical excitation thresholds [24]. The most commonly used of the NF protocols is theta suppression/beta enhancement, usually enhancing sensorimotor rhythm (SMR) simultaneously [15,25]. Studies comparing the effects of NF to stimulant medication found that the effects of NF were at least comparable to stimulant medication in measures of inattention and impulsivity [26,27] and that these effects persisted after medication wash-out only for the group that also received NF [28]. Monastra and colleagues [18] analyzed the empirical evidence of NF in a review applying the guidelines of efficacy concurrently established by the Association of Applied Psychophysiology and Biofeedback and the International Society for Neuronal Regulation. They concluded that NF is “probably an efficacious instrument” for the treatment of ADHD. Arns, De Ridder, Strehl, Breteler, and Coenen [25] highlight that the level of clinical efficacy has been determined to be “efficacious and specific” and, according to Lofthouse, Arnold, Hersch, Hurt, and deBeus [29], “probably efficacious”. In addition, the American Academy of Pediatrics [30] approved NF as “level 1 best support” in an evidence-based treatment for childhood ADHD, which means that there have been studies with acceptable sample sizes showing that NF is safe and effective in the intervention of ADHD symptoms in children although evaluations of NF outside of research trials have been more limited, indicating it is not yet known how laboratory studies translate to real clinical practice.

Nonetheless, the efficacy of NF could vary depending on the type of ADHD presentation, especially considering the differences between the three types of ADHD. With this in mind, it makes sense to analyze the differential effect of an NF intervention on improvement in the different types of presentation, which is the main aim of this current study. One meta-analysis, which included 15 studies [25], found that NF resulted in large and clinically significant effect sizes for inattention and impulsivity and a medium effect size for hyperactivity. 

Bakhshayesh et al. [21] compared clinical and neuropsychological effects of theta/beta training and showed a large effect size for inattention and small to medium effect sizes for hyperactivity and impulsivity, respectively. Considering that the NF effect seems to be greater in inattention and impulsivity, there seem to be different benefits depending on the type of ADHD presentation. 

The meta-analysis by Micouland-Franchi, Geoffroy, Fond, Lopez, Bioulac, and Philip [31] examined the efficacy of NF on overall ADHD symptoms as well as on the inattention and hyperactivity/impulsivity dimensions assessed by parents and teachers, who had no knowledge that their children had received NF. They found an improvement in parent assessments for overall ADHD scores, inattention scores, and hyperactivity/impulsivity scores in NF groups compared to the control groups. For the teacher assessments, improvements were only found for the inattention scores. All of these studies took symptoms into account instead of the specific diagnosis. In the present study, the improvement after the intervention was assessed based on a diagnosis of the children.

The efficacy of NF could also vary depending on the measures analyzed (observed symptoms, performance, or cortical activation). For example, Rossiter [27] observed an improvement in behavioral measures (assessed with a scale for parents) after NF training, but no differences in performance (assessed with the test of variables of attention, TOVA) compared to participants who received medication. Monastra et al. [28] also showed that participants who received NF training exhibited significant improvements in behavioral and neuropsychological measures and an increase in cortical activation assessed by quantified electroencephalography (QEEG).

The aim of this current study was to analyze the differential efficacy of NF training on executive control, cortical activation, and observed symptomatology for the ADHD presentation types. To achieve this, we used three groups: subjects with a diagnosis of ADHD-I, ADHD-HI, and ADHD-C. All groups received an NF intervention based on the classic beta-theta protocol (reduce theta and enhance beta). The working hypothesis was that, although all three groups would show improved performance in the assessed variables (executive control with TOVA, cortical activation with QEEG, and observational symptomatology with the Scale of assessment of attention deficit with hyperactivity (EDAH)), based on previous research [21,31] the gain would differ for each type of presentation and in the different measures or variables evaluated.

## 2. Experimental Section

### 2.1. Participants

The participants in this study were 64 students with ADHD, 22 girls and 42 boys, between 8 and 12 years old (*M* = 9.58; *SD* = 1.11). They were identified at the Child and Adolescent Psychiatric Service of the Central University Hospital of Asturias, according to the *Diagnostic and Statistical Manual of Mental Disorders*, 5th edition [1]. The subjects in the ADHD groups had been diagnosed with ADHD by their child neurologist of reference which allowed the classification of the participants into the three groups according to the ADHD presentation type: ADHD-I (*n* = 15, 6 girls and 9 boys), ADHD-HI (*n* = 11, 6 girls and 5 boys), and ADHD-C (*n* = 38, 10 girls and 28 boys). They all had an IQ of 80 or above (see Table 1), assessed using the Wechsler Intelligence Scale for Children [32] by a specialist psychologist with a master’s degree in education who collaborated with the research group. The presence of a comorbid disorder (learning difficulties, conduct disorder, emotional problems) was used as an exclusion criterion for participation in the study.

Analyses of the participants in this study showed that the sample was homogeneous, with no statistically significant differences in terms of IQ (*p* = 0.666) or age (*p* = 0.515). Differences in terms of gender were significant so this variable was taken as a covariate in subsequent analysis *χ*^2^(1) = 6.25, *p* < 0.012. In addition, 31 participants had pharmacological support (7 ADHD-I, 4 ADHD-HI, and 20 ADHD-C), which was taken as a covariate in the analysis linked to gender.

### 2.2. Instruments

The assessment was carried out at two different times (before and after treatment) using three kinds of measures: assessment of symptoms (EDAH-assessment), assessment of performance (TOVA), and assessment of cortical activation (QEEG). The EEG spectrum was used for the NF intervention.

Scale of assessment of attention deficit with hyperactivity (EDAH) [33]: This scale for parents was used for the assessment of ADHD symptoms [33]. The scale consists of 20 items which provide information about attention deficit (AD; 5 items), hyperactivity–impulsivity (H; 5 items), and conduct disorder (CD; 10 items). The method of response is based on a 4-point Likert-type format, with values between 0 and 3. The Cronbach’s Alpha is high for the total scale (α = 0.929) and the subscales: AD (α = 0.898), H (α = 0.849), and CD (α = 0.899). For the purposes of this study, only the subscales AD and H were considered. Scores over 90% are indicative of attention deficit and/or hyperactivity–impulsivity. 

Test of variables of attention (TOVA) [34]: This test assesses the performance of students over 4 years old. The test is performed on a computer screen where two alternative stimuli are presented during an average of 22.5 min. The target stimulus is a white square with a smaller black square on the upper border, and the non-target stimulus is a white square with a small black square on the lower border. Participants should press a button when the target stimulus appears, but they should not perform any actions when the non-target stimulus appears. Prior to the beginning of the test, the participants had an initial 3-min practice session. The TOVA test produces four main variables: omissions (OM), commissions (COM), response time (RT), and variability (VAR). The instrument has a mean of 100 and a standard deviation of 15. A score of 85 in omissions and response time reflects attention deficit; in commissions, it reflects impulsivity; and in variability, it indicates hyperactivity. Other variables provided by the instrument are the D value (D′) and the ADHD score. D′ is related to hyperactivity symptoms and is a result of the participant’s performance in the test, and the scores increase according to the number of errors. The ADHD score is obtained from the sum of the response time of the first half, D′ of the second half, and the total variability. The ADHD score is indicative of a deficit in executive control when the value is lower than −1.80. 

Quantified electroencephalography (QEEG). The QEEG assessment of cortical activation was used, which provides the levels of cortical activation through the beta/theta ratio. To record the beta/theta ratio, an electrode is placed on the corresponding cortical areas (central area of the cortex (Cz), and left prefrontal area (Fp1) and two more control electrodes are put on the left and right earlobes. The QEEG was administered to each subject, with open eyes, with a duration of 10 min approximately. An electromyogram system (EMG) should be put on the right forearm to control the level of movement. Participants were requested to remain relaxed, without moving, and to concentrate exclusively on the computer screen. A beta/theta ratio lower than 50% at Cz is related to inattention symptoms. For each part, a ratio below 50% at Fp1 is associated with hyperactivity symptoms. [35].

Neurofeedback (EEG spectrum): The intervention in activation levels was enhanced by means of neurofeedback using the EEG spectrum (www.neurocybernetics.com) designed by Howard Lightstone for Neurocybernetics, Inc (Canoga Park, Los Angeles, CA, USA). In accordance with the description included in a recent study [20], the instrument presents two computer screens: one for the professional and the other for the patient. The subject is connected to the apparatus through an EEG preamplifier with wires connected by simple electrodes: signal, ground, and reference. The electrode signal is fixed to the prefrontal area (Fp1) with conductor gel, and the reference and ground electrodes are placed on the earlobes. The instrument takes samples of the EEG signal 256 times per second. The trainer’s software processes the samples of the transformed digital signals and stores, filters, and separates them into various frequency bands, visualizing both the unprocessed signals and the filtered signals on the computer at a rate of 160 samples per second. Brain wave amplitude data at each frequency band are transmitted by the trainer’s computer to the subject’s computer as a game. The trainer monitors the activity of the brain waves and sets the goals, while the patient experiences feedback through the game. 

### 2.3. Design and Data Analysis

We used a quasi-experimental design with three groups (ADHD-I, ADHD-HI, and ADHD-C). All three groups received the neurofeedback intervention. 

Firstly, the means and standard deviations for each variable were analyzed. Subsequently, we analyzed the difference between the pre-treatment and post-treatment scores in the three types of presentation using the Student’s *t* test for related samples. These differences in improvement were analyzed via a multivariate analysis of covariance (MANCOVA) using the gain (the difference between the pre-treatment and post-treatment scores) as a dependent variable and the group (ADHD type of presentation) as an independent variable. Gender and pharmacological support were used as covariables. Once the covariables were removed, post-hoc multiple Scheffé comparisons were performed to determine which groups had significant differences between them. MANCOVAs were performed to look at the pairwise comparisons more deeply, with the three possible pair comparisons: ADHD-HI vs. ADHD-I, ADHD-HI vs. ADHD-C, and ADHD-I vs. ADHD-C. In these three comparisons the gain in the different measures was taken as the dependent variable and gender and pharmacological support as covariables. We used Cohen’s criterion to interpret effect size, which states that the effect is small when *η_p_*^2^ = 0.01 (*d* = 0.20), medium when *η_p_*^2^ = 0.059 (*d* = 0.50), and large when *η_p_*^2^ = 0.138 (*d* = 0.80) [36]. SPSS version 17 was used to perform the statistical analyses. 

### 2.4. Procedure

This study was conducted in accordance with the Helsinki Declaration of the World Medical Association [37] and was approved by the Ethical Committee of the Principality of Asturias (Approval No. CPMP/ICH/135/95, CODE: TDAH-Oviedo). After obtaining parents’ consent, each child was assigned to a reference group according to their diagnosis (ADHD-I, ADHD-HI, and ADHD-C). Then we administered the pre-treatment assessment with QEEG and TOVA.

NF training was carried out for 15 min, 3 days a week, for 3 months (36 sessions). The training began with the EEG spectrum rocket game. In the event that the students could not attend a training session, a make-up class was held in the same week in order to ensure that all the participants received three weekly sessions. After 3 months, all the participants were assessed again with the aforementioned instruments (post-treatment assessment) to evaluate the effects of the intervention. Typical NF interventions in ADHD involve 30–40 sessions, each lasting 30–60 min [21,38]. 

All the assessment tasks (and the intervention process) were coordinated and guided by the same psychologist, who was a member of the research group.

Students with pharmacological support received methylphenidate (one of the most common stimulant medications used to treat ADHD) with the dose set by the child neurologist according to parameters such as weight and height. They started medication after diagnosis at the same time that the NF training was applied. The presence of pharmacological support was taken as a covariate in the specific analysis. The medication was discontinued 1 day before the post-treatment assessment.

## 3. Results

### 3.1. Pre- and Post-treatment Results for the Four Groups

Table 2 gives the data including means and standard deviations for each group of variables (cortical activation with QEEG—central and left prefrontal; executive control with TOVA—omissions, commissions, response time, variability, D′, and ADHD score; and observation with EDAH completed by parents).

### 3.2. Gain after the Intervention

Table 3 shows the gain in each set of variables for the three types of presentation. All three types of presentation showed improvement following the intervention. ADHD-I had significant differences pre-treatment and post-treatment in all variables except hyperactivity measured by EDAH. ADHD-HI showed significant improvement in all variables except RT measured by TOVA. ADHD-C showed significant improvement in all variables with mainly large effect sizes. 

The differences in the gain between the types of presentation were analyzed using MANCOVA (with gender and pharmacological support as covariate variables, the latter being significant as a covariate for *p* ≤ 0.001). The results were statistically significant for all variables except commissions and D prime, measured by TOVA, and AD and H + AD, measured by EDAH (see Table 3). Post-hoc analysis showed that the differences in cortical activation were in Cz between ADHD-HI and ADHD-C (*p* = 0.006) and in Fp1 between ADHD-HI and ADHD-I (*p* = 0.048) as well as between ADHD-I and ADHD-C (*p* = 0.003). 

In performance, differences were significant between ADHD-HI and ADHD-C for omissions (*p* = 0.019), RT (*p* = 0.003), and ADHD score (*p* = 0.020); between ADHD-I and ADHD-C in variability (*p* = 0.005) and ADHD score (*p* = 0.004); and between ADHD-I and ADHD-HI in variability (*p* = 0.041) and RT (*p* = 0.039). 

**Figure 1:**
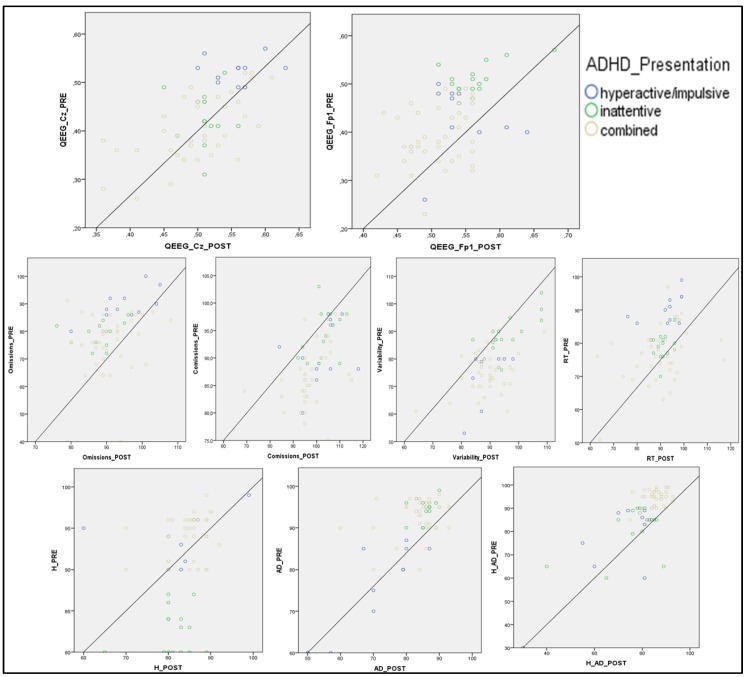
Scatter plot for the variables of cortical activation (Cz and Fp1), execution (omissions, commissions, variability, and RT) and observed symptomatology (H: hyperactivity, AD: attention deficit) considering the three types of presentation. Note: OM: omissions; COM: commissions; VAR: variability; RT: response time; D′: D prime; H: hyperactivity; AD: attention deficit.

Finally, for observation symptomatology, differences were significant in the variable H between ADHD-H and ADHD-I (*p* ≤ 0.001) and between ADHD-C and ADHD-I (*p* ≤ 0.001). Figure 1 provides a visual analysis of the results distribution for paired data in each variable.

### 3.3. Results of Pairwise Group Comparison

In order to examine the pairwise comparisons shown in the post-hoc analysis more deeply, the MANCOVAs were replicated taking the groups in pairs. 

When the ADHD-HI group was compared to the ADHD-I group, differences were statistically significant *F*(11, 12) = 11.261, *p* ≤ 0.001 *η_p_*^2^ = 0.912. The results for each variable are: Cz *p* = 0.022, *η_p_*^2^ = 0.215; Fp1 *p* = 0.004, *η_p_*^2^ = 0.324; omissions *p* = 0.114; commissions *p* = 0.356; variability *p* = 0.005, *η_p_*^2^ = 0.306; RT *p* ≤ 0.001, *η_p_*^2^ = 0.535; D′ *p* = 0.314 and ADHD score *p* = 0.931; H *p* = 0.004, *η_p_*^2^ = 0.313; AD *p* = 0.046, *η_p_*^2^ = 0.168; H + AD *p* = 0.878.

In the case of ADHD-HI and ADHD-C, differences were also statistically significant *F*(11, 35) = 2.952, *p* ≤ 0.007 *η_p_*^2^ = 0.481. Looking at each of the variables, the results are: Cz *p* = 0.003 *η_p_*^2^ = 0.179; Fp1 *p* = 0.712; omissions *p* = 0.009 *η_p_*^2^ = 0.143; commissions *p* = 0.829; variability *p* = 0.806; RT *p* = 0.008 *η_p_*^2^ = 0.146; D′ *p* = 0.423 and ADHD score *p* = 0.007 *η_p_*^2^ = 0.152. H *p* = 0.318; AD *p* = 0.215; H + AD *p* = 0.228.

Finally, the comparison of ADHD-I and ADHD-C was statistically significant *F* (11, 39) = 8.413, *p* ≤ 0.001 *η_p_*^2^ = 0.704. The results for each variable are: Cz *p* = 0.375 Fp1 *p* ≤ 0.001 *η_p_*^2^ = 0.253; omissions *p* = 0.039 *η_p_*^2^ = 0.084; commissions *p* = 0.412; variability *p* = 0.001 *η_p_*^2^ = 0.206; RT *p* = 0.745; D′ *p* = 0.037 η_p_^2^ = 0.086 and ADHD score *p* ≤ 0.001 *η_p_*^2^ = 0.250. H *p* ≤ 0.001 *η_p_*^2^ = 0.339; AD *p* = 0.964; H + AD *p* = 0.040 *η_p_*^2^ = 0.083.

## 4. Discussion

The main goal of this work was to analyze the differential effect of NF on the three presentation types of ADHD. The design was based on analyzing the benefits of this type of intervention in three areas: cortical activation, performance, and observed symptomatology. 

Firstly, the results showed that, in general, the three presentation types showed improvements in all three areas following the intervention, although these differences were not statistically significant in all cases. In cortical activation, the three types showed a positive improvement. The direction of the differences indicated that this improvement was greater in ADHD-C and ADHD-HI than in ADHD-I in Fp1. In Cz, the improvement was better in ADHD-C and ADHC-I than in ADHD-HI. However, if we focus on effect size, this is largest in ADHD-C followed by ADHD-HI for Cz and in ADHD-C followed by ADHD-I for Fp1. Thus, the results with regard to cortical activation suggest that ADHD-C and ADHD-HI benefit most from the intervention. This may be related to the fact that ADHD-I does not have a major deficit in Fp1, and a similar case occurs for ADHD-HI in Cz [35]. In any case, the ADHD-I group showed good improvement in activation in the Fp1 area with a high effect size compared to the Cz area. The intervention with NF was carried out at the Fp1 point and the results showed that the intervention in this area had a greater effect in the cortical activation of Fp1 than on Cz (as we can see in the three types of presentation where the effect size is lower in Cz compared with Fp1).

In executive control, the profile in the six variables was similar. ADHD-C and ADHD-HI demonstrated better progression than ADHD-I when looking at mean scores. ADHD-C improved more than ADHD-HI except in commissions (the variable associated with impulsivity). Analyzing the progression of each type of presentation, the effect size reflected that the improvement was greater in ADHD-I for omissions and RT (variables related to attention). In the case of ADHD-HI the effect size was greater for variability and D′, and in ADHD-C it was greater for variability and the ADHD score (obtained from the set of RT, variability, and D prime). Given that the Fp1 point (where the intervention was done) is related to inhibition of behavior, hyperactivity, and impulsivity, it is logical that the ADHD-C and ADHD-HI groups improved more than ADHD-I, mainly given that ADHD-I has no major problems associated with impulsivity or hyperactivity, and, hence, the range of improvement is higher for the ADHD-HI and ADHD-C groups. However, children with the inattentive type improved in their RTs and omissions, aspects where children with this specific presentation have more difficulties, and exhibited higher effect sizes in these two variables, reflecting that the intervention in Fp1 also has effects on variables related to inattention. 

In the case of observed symptomatology measured with EDAH, parents reported good progression after the intervention in the three types of presentation. Again, parents of students with ADHD-C reported better improvement. Parents of students with ADHD-I saw better progression in inattentive symptoms, and parents of ADHD-HI students reported greater improvement in hyperactive impulsivity, as expected. 

In short, we analyzed differences in the gain (the improvement in scores) in the presentation types of ADHD. The conclusion is that the presentation types have such different profiles of activation and performance that the pre-to-post-test progression varies significantly. The effect size of the pairwise group comparison showed that the ADHD-HI and ADHD-I groups differed mainly in the gain in cortical activation and observed symptomatology but not especially in performance, where the differences were also significant for variability and RT. The ADHD-HI and ADHD-C groups showed greater differences in the gain in performance but the progression of these two types of presentation was very similar without differences in the gain in observed symptomatology. On the other hand, the ADHD-I and ADHD-C groups showed differences in the three kinds of measures: activation (Fp1), performance (omissions, variability, D prime and ADHD score), and observed symptomatology (hyperactive impulsivity symptoms and inattentive symptoms). 

In general, one conclusion that we can draw is that although ADHD-C represents the combination of symptoms, this group demonstrates better improvement after the intervention. This could be related to the fact of the initial scores being lower than the other types so there was more room for improvement. Furthermore, the intervention was focused on the Fp1 area, but the improvements in performance were in the variables associated not only with hyperactivity and impulsivity but also the variables associated with inattention (e.g., the ADHD-I group shows benefits in RT). The improvement produced in cortical activation had an effect on performance and on the observed symptoms. As in studies such as Monastra et al. [28], we saw that the intervention had an effect on the three kinds of measures: cortical activation, performance, and observed symptomatology. 

These results are consistent with previous research in which NF intervention had a positive effect on ADHD symptoms [15,25]. Furthermore, as Fuchs and colleagues [26] pointed out, NF has an effect on measurements of inattention and impulsivity. This is more significant when noting that around 40% to 60% of cases of ADHD persist into adolescence and adulthood [6] and that stimulant medication is effective in reducing ADHD symptoms in only 70%–80% of cases [9]. It highlights the need to include different treatments that allow the reduction of symptoms and help students to face the difficulties the disorder poses. 

The meta-analysis from Hodgson et al. [11] showed that interventions with NF were generally more efficacious for girls and least efficacious for ADHD-C. It is possible that this depends on the intervention protocol. An NF intervention situated in the Fp1 area produces a marked improvement in hyperactivity and impulsivity over inattention deficit. This would make sense in relation to other studies [35,39], in which the authors concluded that the Fp1 area is more affected in ADHD-HI and ADHD-C and that the Cz area is more affected in ADHD-I and ADHD-C. Given that the intervention was carried out in the Fp1 area, in which students with ADHD-HI and ADHD-C have more difficulties, it is understandable that they showed a greater improvement after intervention. 

One implication of these results is that the intervention must be adapted to the specific profile of the students [40,41]. It is necessary to analyze the improvements of ADHD in different points or cortical areas in the future to determine the most effective intervention protocol for each diagnosis. This would produce better results from the intervention in a shorter time. Finally, we must consider some of this study’s limitations, such as the absence of a control group, the small sample size in each group, and the selection of the students who were chosen to have this specific NF intervention based on their parents’ wishes. Furthermore, as the intervention program was applied for only 15 min, it would be interesting to study the benefits of an intervention with 30-min periods and maybe the difference between the effects of these two durations. It would allow the intervention to be better adjusted and the establishment of a training protocol. In this regard, according to Duric and colleagues [17], there is no standard recommendation for the number, duration, or frequency of sessions when these types of protocols are administered; hence, it is a challenge in this area of research.

## Figures and Tables

**Table 1:** Means and Standard Deviations of IQs and Age of the Groups.

Groups		IQ		Age		Gender F/M
	*n*	*M*	*SD*	*M*	*SD*	
ADHD-I	15	96.67	13.947	9.667	1.128	6/9
ADHD-HI	11	97.45	9.095	9.883	1.456	6/5
ADHD-C	38	99.47	10.118	9.458	1.005	10/28
Total	64	98.47	10.860	9.580	1.113	22/42

ADHD: attention deficit with hyperactivity disorder, IQ: intelligence quotient, M: mean, SD: standard deviation.

**Table 2:** Pre- and Post-treatment means and standard deviations for cortical activation, execution, and observation.

	**ADHD-I** **(*n* = 15)**		**ADHD-HI** **(*n* = 11)**		**ADHD-C** **(*n* = 38)**		**Total** **(*n* = 64)**	
	**Pre** ***M*(*SD*)**	**Post** ***M*(*SD*)**	**Pre** ***M*(*SD*)**	**Post** ***M*(*SD*)**	**Pre** ***M*(*SD*)**	**Post** ***M*(*SD*)**	**Pre** ***M*(*SD*)**	**Post** ***M*(*SD*)**
Cz	0.43 (0.05)	0.51 (0.02)	0.52 (0.02)	0.55 (0.03)	0.40 (0.06)	0.49 (0.06)	0.43 (0.07)	0.51 (0.05)
Fp1	0.51 (0.02)	0.56 (0.04)	0.43 (0.06)	0.54 (0.04)	0.39 (0.05)	0.50 (0.04)	0.42 (0.07)	0.52 (0.04)
OM	79.20 (4.17)	88.13 (5.08)	89.64 (5.51)	95.00 (7.29)	75.97 (11.24)	91.05 (6.53)	79.08 (10.40)	91.05 (6.63)
COM	93.66 (4.45)	102.73 (5.86)	91.00 (5.77)	102.27 (9.28)	86.55 (5.36)	96.84 (8.40)	88.98 (6.00)	99.16 (8.41)
VAR	89.47 (6.49)	96.13 (7.22)	74.82 (9.23)	89.00 (5.13)	74.71 (6.64)	88.92 (7.34)	78.19 (9.40)	90.62 (7.54)
RT	79.13 (3.81)	91.00 (3.20)	90.36 (4.27)	92.55 (7.69)	76.76 (8.50)	90.29 (11.15)	79.66 (8.58)	90.84 (9.24)
D′	−1.15 (0.44)	−0.64 (.34)	−1.32 (0.86)	−0.57 (0.70)	−1.78 (0.62)	−0.81 (.54)	−1.55 (0.68)	−0.73 (.53)
ADHD score	−2.92 (0.64)	−1.70 (0.87)	−2.84 (1.32)	−1.54 (1.34)	−4.45 (1.44)	−1.94 (0.86)	−3.82 (1.55)	−1.81 (0.96)
H	82.53 (2.72)	81.27 (5.32)	93.09 (3.14)	82.00 (9.14)	93.89 (2.72)	84.79 (4.93)	91.09 (5.52)	83.48 (6.03)
AD	94.53 (2.64)	85.60 (2.82)	75.18 (10.82)	69.91 (12.76)	92.76 (4.44)	83.76 (6.66)	90.16 (8.93)	81.81 (9.17)
H + AD	81.53 (10.07)	76.73 (11.84)	76.18 (18.30)	70.36 (16.28)	94.00 (3.54)	84.92 (4.34)	88.02 (11.79)	80.50 (10.83)

ADHD-I: inattentive type; ADHD-HI: Hyperactive–impulsive type; ADHD-C: combined type; M: mean, SD: standard deviation.

**Table 3:** Student’s *t*-Test and MANCOVA analyzing the gain and the differences between the types of presentation.

	**ADHD-I** **(*n* = 15)**		**ADHD-HI** **(*n* = 11)**		**ADHD-C** **(*n* = 38)**				
	***t*(14)**	***d***	***t*(10)**	***d***	***t*(37)**	***d***	***F*(2, 59)**	***η_p_*^2^**	**Post-hoc**
Cz	−5.01 ***	0.66	−2.41 *	1.08	10.38 ***	2.41	5.39 **	0.155	C > HI
Fp1	−6.06 ***	2.29	−4.54 **	2.03	11.62 ***	2.7	7.18 **	0.196	C&HI > I
OM	−6.08 ***	2.3	−3.46 **	1.55	7.93 ***	1.84	5.75 **	0. 163	C > HI
COM	−6.03 ***	2.28	−4.02 **	1.8	7.81 ***	1.81	0.25	0.009	
VAR	−4.79 ***	1.81	−6.28 ***	2.81	11.17 ***	2.6	6.76 **	0.186	C&HI > I
RT	−10.43 ***	3.94	−1.10	0.49	7.49 ***	1.74	5.10 **	0.147	C&I > HI
D′	−3.22 **	1.22	−5.81 ***	2.6	7.78 ***	1.81	2.59	0.081	
ADHD score	−6.16 ***	2.33	−2.93 *	1.31	12.01 ***	2.79	9.47 ***	0.243	C > HI&I
H	0.87	0.33	−4.26 **	2.02	11.35 ***	2.64	12.37 ***	0.296	HI&C > I
AD	11.68 ***	4.41	−3.03 *	1.36	7.96 ***	1.85	1.30	0.042	
H + AD	1.67 *	0.63	−1.74	0.78	12.56 ***	2.92	2.14	0.068	

ADHD-I: inattentive type; ADHD-HI: hyperactive–impulsive type; ADHD-C: combined type; OM: omissions; COM: commissions; VAR: variability; RT: response time; D′: D prime; H: hyperactivity; AD: attention deficit. *** *p* ≤ 0.001; ** *p* ≤ 0.01; * *p* ≤ 0.05.
